# Functional investigation of the *RHD* gene promoter: Molecular changes are rarely responsible for variant D phenotype in Thai donors

**DOI:** 10.1111/trf.70155

**Published:** 2026-03-07

**Authors:** Caroline Bénech, Pornlada Nuchnoi, Yann Fichou

**Affiliations:** ^1^ Univ Brest, Inserm, EFS UMR1078, GGB Brest France; ^2^ Department of Clinical Microscopy, Faculty of Medical Technology Mahidol University Salaya Thailand; ^3^ Center for Research Innovation and Biomedical Informatics, Faculty of Medical Technology Mahidol University Bangkok Thailand

**Keywords:** phenotype, promoter, RH antigen, *RH* genes, variant

## Abstract

**Background:**

While modern technology is increasingly available to identify efficiently the molecular alterations responsible for variant phenotype, a subset of samples still escapes those analyses. In addition to the promoter region, two intronic regions containing GATA1 binding sites within the *RHD* gene locus were recently shown to modulate the transcriptional activity of the gene. We then thought to explore these regions in a subset of samples with unresolved genotypes.

**Study Design and Methods:**

Thirty‐three Thai donor samples (weak D, *n* = 10; D‐negative, 23) were selected for molecular analysis by conventional Sanger sequencing. In parallel, a novel functional assay was designed for investigating the potential effect of variants within the *RHD* gene promoter. Three variants previously reported, that is, c.1–115A>C, c.1–110A>C, and c.1–83G>T, were tested by this approach.

**Results:**

No variant was identified in the 33 samples, suggesting that the molecular basis of the respective phenotypes remains to be resolved. Interestingly, beyond the ~200 base pair (bp) promoter region commonly described, another ~300 bp region located upstream appears to contain elements that positively regulate the transcriptional activity of the *RHD* gene. Also, the deleterious effect of the c.1–110A>C variant was confirmed, conversely to the two other variants, which did not show any functional alteration.

**Discussion:**

Regulation of *RH* gene expression is likely to involve other levels of complexity that remain to be identified. Future investigations are necessary to characterize those critical genomic regions that potentially carry rare variants responsible for weakening or abolishment of D antigen expression.

**Abbreviations**
gDNA: genomic deoxyribonucleic acid
ISBT: International Society of Blood Transfusion
PCR: polymerase chain reaction
SNV: single nucleotide variant
SV: structural variant

## 1 INTRODUCTION

To date, hundreds of variants/alleles have been reported in the *RH* genes, the genes encoding the proteins carrying the RH blood group antigens (ISBT Red Cell Immunogenetics and Blood Group Terminology Working Party). These variants are known to promote the disruption of the molecular and/or cellular mechanism(s) involved in the regulation of gene expression, thus resulting in a variant RH phenotype characterized by a quantitative and/or a qualitative defect.^1^ The D antigen (RH1) has long been considered to be the most clinically relevant RH antigen. Several reports have shown that missense single nucleotide variants (SNVs), which are responsible for an amino acid change at the protein level, can induce a partial phenotype by modifying the extracellular moiety of the D antigen.^2^, ^3^ In addition, protein structure, as well as intra‐ and intermolecular interactions with protein partners, may be impaired by those missense variants thus contributing to reduce the amount of RhD protein and to weaken D antigen expression.^2^, ^3^, ^4^ Our group and others have also shown that several SNVs are causative for a variant phenotype by altering splicing.^5^, ^6^, ^7^, ^8^, ^9^, ^10^, ^11^ Overall, the spectrum of functional defects induced by *RH* gene variants and resulting in variant D phenotype is broad, and has been documented in many reports.

Nowadays, standard and modern techniques are commonly available in reference molecular immunohematology laboratories to identify efficiently the genetic determinant(s) of the variant phenotype.^12^, ^13^, ^14^ However, recent publications still commonly report samples of various origins exhibiting a well‐characterized variant D or D‐negative phenotype, but that do not carry any SNVs and/or structural variants (SVs) in the gene regions typically investigated by conventional and advanced approaches (i.e., coding DNA regions).^15^, ^16^, ^17^, ^18^, ^19^, ^20^, ^21^, ^22^, ^23^, ^24^, ^25^ This observation suggests that additional molecular alterations affecting other mechanisms are likely to be discovered in alternative regions, and therefore to be responsible for the phenotype.

Recently, an elegant, comprehensive study using bioinformatic predictions reported the identification of candidate regions in both the promoter and introns of the *RHD* gene that are thought to bind GATA1,^26^ a transcription factor that is critical for erythropoiesis. The authors postulated that variants within these regions may be responsible for variant phenotype. Interestingly, a novel c.1–110A>C variant, which is located at the 3′‐end of a GATA1 binding site in the proximal promoter, (1) was found in a single donor presenting with a weak D phenotype characterized by routine serological typing, and (2) was demonstrated to reduce significantly the activity of the promoter region by functional analysis (~73% with c.1–110A>C vs. 100% in wild‐type conditions). The results of this study prompted us to explore those regions of interest in a subset of samples previously investigated in our laboratory that were negative for *RHD* variant screening, that is, no variant identified, while presenting with either a variant D or D‐negative phenotype. We also thought to develop a novel robust assay for (1) reinvestigating the functional regions of the *RH* gene promoters and analyzing potential future variants; and (2) confirming the effect of those SNVs reported previously. Here we report our findings.

## 2 MATERIALS AND METHODS

### 2.1 DNA samples

Thirty‐three Thai donor samples with undetermined molecular cause of weak/partial D (*n* = 10) or D‐negative (23) phenotype were selected for molecular analysis of the three *RHD* gene candidate regions (Table S1).^22^, ^24^

### 2.2 *RHD* gene targets and Sanger sequencing

Three regulatory regions located within the promoter, intron 1, and intron 2 were analyzed by sequencing in each sample (Table 1).^26^ Briefly, PCR amplifications were carried out using a commercial reagent (HotStarTaq Master Mix, Qiagen, Courtaboeuf, France) with 0.4 μM of both forward and reverse primers and 50–100 ng genomic DNA (gDNA) as a template in the following conditions: 95°C, 15 min followed by 35 cycles at 95°C, 30 s; 60°C, 30 s; 72°C, 30 s; and a final extension step at 72°C, 10 min. After enzymatic treatment (ExoSAP‐IT PCR Clean Up Product Reagent, Thermo Fisher Scientific, Courtaboeuf, France), cycle sequencing reaction was performed in the conditions recommended by the manufacturer (BigDye Terminator v1.1 Cycle Sequencing Kit, Thermo Fisher Scientific), and the purified products were sequenced by capillary electrophoresis (SeqStudio Flex Genetic Analyzer, Thermo Fisher Scientific).

**TABLE 1:** Primers for PCR amplification and sequencing of three *RHD* gene targets.

Target	PCR product size	Genomic coordinates^a^ (hg38)	Primer ID	Primer sequence (5′ → 3′)
Promoter	853 bp	Chr1:25,271,633–25,272,485	RHD_pro1_F	CACATGGATGGGAGCACAGG
			RH_prom_R^b^	TACTTGAGGGCTTGAGGGAGC
Intron 1	532 bp	Chr1:25,279,680–25,280,211	RH_int1ATACseq_F^b^	TGTGTGCCTGCTGTTACAAC
			RH_int1ATACseq_R^b^	CCTTCCTCCCCAAGGCTCTAC
Intron 2	597 bp	Chr1:25,284,849–25,285,445	RH_int2ATACseq_F^b^	TCTCTGTCTAGCACCAGTGCTGTG
			RH_int2ATACseq_R^b^	GAATGTCTCTCAGGCTGGACGC

*Note*: Homologous regions in *RHCE* are amplified and sequenced simultaneously for “Intron 1” and “Intron 2” targets.

Abbreviation: bp, base pairs.

^a^ In the *RHD* gene.

^b^ Primer sequences adapted from RHD_prom_HindIII_R, KpnI_RH_int1ATACseq_F, KpnI_RH_int1ATACseq_R, KpnI_RH_int2ATACseq_F, and KpnI_RH_int2ATACseq_R, respectively.^26^

### 2.3 Plasmid construct and site‐directed mutagenesis

For homogeneity in normalization, a plasmid construct carrying both 1/ a reporter gene under the control of a reference promoter and 2/ another reporter gene controlled by a candidate promoter of interest was engineered. To this aim, we generated the pDP‐Empty vector (Appendix S1) and created a series of plasmid constructs containing the *RHD* promoter region with various lengths (Appendix S1; pDP‐RH208, pDP‐RH508, pDP‐RH1208, and pDP‐RH1508).

The effect of three different variants was tested on *RHD* promoter activity: c.1–110A>C,^26^ as well as c.1–115A>C and c.1–83G>T.^27^ The pDP‐RH208 and pDP‐RH508 plasmid constructs containing the variants were generated by site‐directed mutagenesis (QuikChange II XL Site‐Directed Mutagenesis Kit, Agilent Technologies, Les Ulis, France) with the respective corresponding primers (Table S2) before extraction and purification (NucleoBond Xtra Midi kit, Macherey‐Nagel).

### 2.4 Cell culture, transfection and dual‐luciferase assay

K562 cells deficient for the expression of the *RHD* gene, that is, K562^*RHD*−/−^,^10^ were cultured in a selective medium consisting of 0.5 mg/mL Zeocin (Thermo Scientific) in IMDM with L‐Glutamine and HEPES (Lonza, Colmar, France) supplemented with 10% fetal bovine serum (FBS, Lonza), as described before.^10^

Plasmids were introduced into K562^*RHD*−/−^ cells using the nucleofection technology (Amaxa Nucleofector II Device, Lonza) in the conditions recommended by the manufacturer (Cell Line Nucleofector Kit V, Lonza). Briefly, 2 μg of plasmids were mixed with K562^*RHD*−/−^ cells (1 × 10^6^) resuspended in 100 μL of supplemented solution (Nucleofector Solution V, Lonza), electroporated (Amaxa Nucleofector II Device using program T‐016, Lonza) in the manufacturer's conditions and directly seeded into 6‐well plate in 2 mL of IMDM (Lonza) supplemented with 10% FBS (Lonza). After 24 h in culture, K562^*RHD*−/−^ cells were washed twice in PBS 1X and treated with commercial reagents (Dual‐Luciferase Reporter Assay System, Promega, Charbonnières‐les‐Bains, France) as recommended in the manufacturer's protocol before sequential luciferase activity measurement of both reporter genes on a microplate reader (Varioskan, Thermo Fisher Scientific). All experiments were repeated independently four times.

## 3 RESULTS

### 3.1 Variants are uncommon in the regions containing the putative GATA1 binding sites

Three regulatory regions of interest identified in a previous report^26^ were sequenced in all 33 DNA samples from Thai donors expressing either a weak/partial D or a D‐negative phenotype in whom no variant could be found by standard molecular techniques. Excluding the specific nucleotide positions that are due to the homology in the target regions of the *RH* genes, no variant was identified. This result suggests that the molecular determinants of the phenotype observed in these donors remain to be discovered.

### 3.2 A ~500 bp promoter region is necessary and sufficient for optimal *RH* promoter activity

**FIGURE 1:**
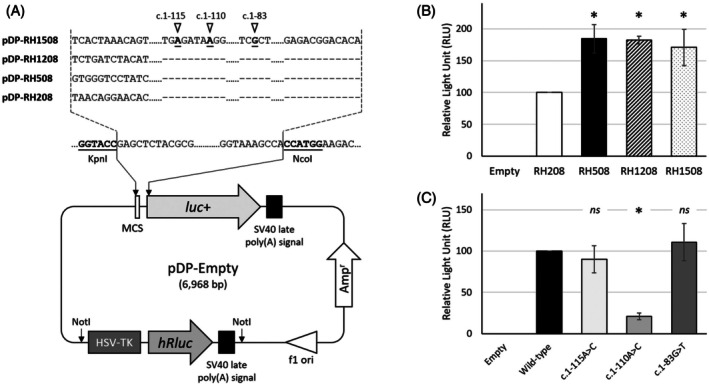
Design and results of the dual‐luciferase reporter assay. (A) Schematic representation of the novel pDP‐empty plasmid vector containing both the *hRluc* and *luc*+ reporter genes under the control of the HSV‐TK promoter and promoter of interest, respectively (see Appendix S1 for details); Amp^r^, β‐lactamase gene; MCS, multiple cloning site; pDP‐RH1508, pDP‐RH1208, pDP‐RH508, and pDP‐RH208 respectively design the pDP‐Empty‐based plasmid constructs containing various length of the *RHD* promoter region; c.1–115, c.1–110, and c.1–83 indicate the three positions of interest that were mutated for investigation by the functional assay. Relative transcriptional activity with (B) various length of the *RHD* promoter (reference: pDP‐RH208; RLU = 100), and (C) three variants in the *RHD* promoter region (reference: pDP‐RH508; RLU = 100); Fisher test (*N* = 4): * *p* < 10^−4^, *ns*: not significant.

While, from our experience, normalizing quantitatively the data of a dual luciferase assay may be commonly challenged by the methodology, but also in order to get novel insights into *RH* promoter activity and to further characterize the promoter region, we thought to design a plasmid construct that brings together two reporter genes, that is, *Renilla* Luciferease Reporter Gene (*hRluc*) and *Firefly* Luciferase Reporter Gene (*luc*+) (see Section 2, and Appendix S1). These genes are respectively under the control of the HSV‐thymidine kinase (TK) promoter, which activity is relatively weak; and four *RHD* promoter constructs of interest that differ in length: 208 (i.e., in the range of the minimal promoter previously defined^27^, ^28^), 508, 1208, and 1508 base pairs upstream the reference 5′‐UTR of the *RHD* gene (Ensembl ID: ENST00000328664.9; see Section 2, and Appendix S1), defining respectively the pDP‐RH208, pDP‐RH508, pDP‐RH1208, and pDP‐RH1508 plasmid constructs (Figure 1A).

Functional analysis by dual luciferase assay clearly showed that, in our conditions, the shortest promoter (i.e., RH208) is less active than the three other constructs (Figure 1B; RH508: × 1.84; RH1208: × 1.83; RH1508: × 1.71, respectively, relatively to the activity observed with pDP‐RH208). These results suggest that the pDP‐RH508 promoter construct is necessary and sufficient to yield the optimal promoter activity, and further indicate that the region spanning ~200–500 bp upstream the 5′‐UTR of the *RHD* gene contains at least one regulatory element that enhances the transcriptional activity. For commodity, the next experiments were carried out with the pDP‐RH508 plasmid construct as the reference.

### 3.3 The c.1–110A>C variant significantly lowers *RH* promoter activity

We then thought to test, in our model, those variants of interest that have been shown to be deleterious to various extents for *RH* promoter activity: c.1–115A>C found in a patient exhibiting variable serologic reactions with mixed‐field pattern by routine typing^27^; and c.1–110A>C defining a novel *DEL* allele recently reported in a donor.^26^ In addition, because *RHD* and *RHCE* promoter regions display ~99% sequence similarity over 1.5 kb, we speculate that a variant affecting the activity of one promoter may likely alter similarly the activity of the other promoter. Then, the c.1–83G>T variant,^27^ which was shown to downregulate *RHCE* promoter activity, was included in our test on an *RHD* promoter background. Overall, a total of three different variants were tested using pDP‐RH508 as the background wild‐type plasmid construct.

The c.1–110A>C variant was clearly confirmed to decrease importantly the activity of the *RHD* promoter (Figure 1C, 20.9% of the activity in wild‐type conditions), thus confirming the previous data.^26^ Conversely, neither c.1–115A>C nor c.1–83G>T was shown to alter significantly *RHD* promoter function (Figure 1C: 90.2% and 110.8% of the activity observed in wild‐type conditions, respectively), thus challenging the data of the previous report. To further reinforce our findings, the same variants were subsequently investigated in another series of experiments by using the shorter pDP‐RH208 construct, and interestingly yielded comparable results (Figure S1: 17.7%, 82.7%, and 131.7%, for c.1–110A>C, c.1–115A>C, and c.1–83G>T, respectively). This latter result suggests that those variants alter the activity of the *RHD* promoter independently of its length.

## 4 DISCUSSION

### 4.1 Deleterious variants in the *RHD* promoter remain rare

Gene promoters are critical regions for the regulation of gene expression. While the pivotal report in the field addressed that question at an early stage,^29^ the promoter region of the *RH* genes is rarely investigated. Nevertheless, a few variants have been shown to be responsible for variant phenotype in both genes. In this report, we sought to investigate the potential presence of causative variants in the promoter, as well as in two intronic regulatory regions recently reported,^26^ in a subset of 33 samples carrying at least one copy of an appearing “wild‐type” *RHD* allele after conventional molecular analysis, while presenting with either a weak D or a D‐negative antigen expression. In a total of 99 sequenced regions (i.e., 3 targeted loci × 33 samples), not a single variant could be found. While the molecular defects responsible for the respective phenotypes remain to be found in these individuals, these results suggest importantly that other mechanisms and genomic regions of interest remain to be investigated and identified.

### 4.2 Functional regions remain to be identified within the *RH* promoters

In an effort to replicate, and potentially to confirm, the results of the functional analysis carried out in previous studies, we designed a novel assay by engineering a plasmid construct carrying both the reference and target promoters driving the expression of reporter genes, thus ensuring the reinforcement of the fluorescence measurement normalization. Our data clearly suggest that, in addition to a minimal promoter of ~200 bp in length previously reported,^27^, ^28^, ^30^ important additional regulatory elements appear to be located within the upstream ~300‐bp region (Figure 1B). Additional experiments will be necessary to identify and characterize those regions at the functional level, and our model may likely be convenient to this aim.

### 4.3 The c.1–110A>C variant, but not c.1–83G>T and c.1–115A>C, may be responsible for weakened D antigen expression

In accordance with McGowan and colleagues, we showed that c.1–110A>C decreases significantly the transcriptional activity of the *RHD* promoter, even at a much lower level, that is, –81% in our model versus −27% in the previous report.^26^ Whereas the reason for this difference may be explained by the nature of the respective functional assays, this observation prompted us to conclude similarly that this variant is likely to be functionally responsible for the DEL phenotype previously reported in a donor.^26^

Conversely, the c.1–83G>T variant has no significant effect in our model, while a −43% decrease in the transcriptional activity was observed by Fennell and collaborators.^27^ The reason for this discrepancy is unclear. Although it is worth mentioning that neither the plasmid nor the promoter background is the same in our respective studies, the previous protocol used co‐transfection for normalization, which is known to result in a broad range of variability in the results.^31^ While our model was engineered for improving significantly the normalization of fluorescence intensity, we hypothesize that our data are likely to reflect more closely the functional impact of the variant in physiological conditions, and conclude that c.1–83G>T is not deleterious for *RH* promoter activity.

The conclusion regarding the functional defect induced by c.1–115A>C variant appears to be more challenging. Indeed, although a 10% decrease in the transcriptional activity was observed in our model, this drop is not statistically significant. In a previous work, a limited effect of the variant was shown (i.e., –4%);^26^ while another study supported its responsibility for explaining the variable observations in serologic D typing (i.e., –17%).^27^ Therefore, although we cannot definitely conclude that c.1–115A>C significantly alters the promoter activity from our observations, the unambiguous causality of this variant is still unclear.

It is finally interesting to note that PromoterAI, a contemporary tool for predicting promoter variants that alter gene expression,^32^ suggests that c.1–110A>C is likely to impair the transcriptional activity of the *RHD* promoter (Promoter AI score: −0.29 [range: −1 to 1; 0: no activity]), while c.1–83G>T and c.1–115A>C are not (scores: 0.04 and −0.04, respectively), thus correlating with the results of our functional analysis. A novel variant, that is, c.1–111A>G, located within the paralogous GATA1 binding site of the *RHCE* gene, was recently reported at the homozygous state in two Chinese patients presenting with the rare D‐‐ phenotype.^33^ By using a functional assay, the authors demonstrated that this variant markedly reduced the transcriptional activity of the *RHCE* promoter, thus being responsible for the downregulation of the expression of the C and e antigens. The score obtained by PromoterAI for this latter variant is −0.37, in agreement with the decrease of gene expression reported in the study.^33^ While variants in *RH* promoter regions are rarely observed, there is no doubt that *in silico* tools, such as PromoterAI, may be helpful in the characterization of functionally deleterious variants in the future, as recently shown for splicing variants.^34^

### 4.4 Web resources

ISBT reference website, Red Cell Immunogenetics and Blood Group Terminology Working Party; (ISBT 004) RHD blood group alleles v6.4 31‐JUL‐2023: https://www.isbtweb.org/resource/004rhd.html.

## FUNDING INFORMATION

This work was supported by the Etablissement français du sang (EFS) Bretagne.

## CONFLICT OF INTEREST STATEMENT

The authors have disclosed no conflicts of interest.

## Supporting information


**APPENDIX S1.** Supporting information.

## Data Availability

The data that support the findings of this study are available from the corresponding author upon reasonable request.
