# Supplementary material for: Functional investigation of the RHD gene promoter: Molecular changes are rarely responsible for variant D phenotype in Thai donors
Source: Transfusion. 2026 Mar 7;66(5):957–63. doi: 10.1111/trf.70155 (PMC13158328; doi:10.1111/trf.70155)
Supplement: Supplementary file 1 — APPENDIX S1. Supporting information. [file TRF-66-957-s001.pdf]

SUPPLEMENTARY MATERIALS AND METHODS

Basic dual-promoter, reporter plasmid construct

Because, in our hands, normalization of gene-reporter activity has long shown variability from one experiment to another, and even inconsistency in some cases, in cell-based assay when using plasmid cotransfection, thus challenging our findings, we sought to engineer a single plasmid construct embedding both a reference gene and a target gene under the respective control of their own promoter. To this aim, a region of the commercial pGL4.74[*hRluc*/TK] Vector (Promega, Charbonnières-les-Bains, France) including 1/ the HSV-TK promoter, 2/ the *hRluc* reporter gene, and 3/ the SV40 late poly(A) signal, for a total of 2,142 base pairs (bp), was amplified using a high-fidelity PCR enzyme (PrimeSTAR GXL DNA Polymerase, TaKaRa, Saint-Germain-en-Laye, France) with 0.3 µM of the RVp3br_NotI_F and RVp4br_NotI_R primers (Table S1), which both contain a NotI restriction site (GCGGCCGC), in the conditions recommended by the manufacturer ($T°_{annealing}$ = 60°C). The PCR product (= insert) was visualized on a 1% agarose gel stained with ethidium bromide and extracted with a commercial kit (NucleoSpin Gel and PCR Clean-up, Macherey-Nagel). In parallel, 1 µg pGL3-Basic Vector (Promega) was digested with 10 U NotI restriction enzyme (New England Biolabs, Evry-Courcouronnes, France) at 37°C for 1 h (= linearized vector), loaded onto a 1% agarose gel stained with ethidium bromide and then extracted and purified as described above.

The insert was subcloned within the linearized vector by homologous recombination (In-Fusion HD Cloning Kit, TaKaRa) at 50°C for 15 min, and 1.5 µL cloning product was transformed into 25 µL competent cells (Stellar Competent Cells, TaKaRa), which were cultured in selective conditions (LB Agar + 50 µg/mL ampicillin) at 37°C, overnight. The colonies were cultured in selective conditions (LB liquid medium + 50 µg/mL ampicillin) at 37°C, 225 rpm, overnight, and the bacteria suspension were pelleted for plasmid miniprep using

a commercial kit (NucleoSpin Plasmid Mini kit, Macherey-Nagel). The identity of all regions of interest (i.e. promoter region, reporter genes, multiple cloning site) was controlled by Sanger sequencing before medium-scale production of the selected plasmid construct and extraction (NucleoBond Xtra Midi kit, Macherey-Nagel). The so-called novel plasmid construct "pDP-Empty" (Figure 1, 6,968 bp) serves 1/ as a control for normalizing the background fluorescence due to the *luc*+ reporter gene in the functional assay, and 2/ as a template for subcloning the promoter regions of interest.

***RHD* promoter subcloning**

The pDP-Empty vector was double-digested with both the KpnI and NcoI restriction enzymes (New England Biolabs, Evry-Courcouronnes, France) at 37°C for 1 h, followed by an inactivation step at 80°C for 20 min. The product was loaded onto a 1% agarose gel stained with ethidium bromide and the largest band (~6.9 kb) was then extracted and purified as described above (= linearized pDP-Empty vector).

In parallel, by using the +1 transcription start site (TSS) as the reference (Ensembl StableID: ENST00000328664.9), four *RHD* promoter constructs (i.e. upstream the TSS) differing in length were chosen for being tested by the functional assay, accounting for 208 (which is comparable to the promoter sequence used in previous reports [Suganuma, 2005; Fennell, 2017]), 508, 1,208, and 1,508 bp. The sequences of interest were synthesized by a company (GenScript Biotech, Rijswijk, Netherlands) and served as templates for PCR amplification with a high-fidelity PCR enzyme (PrimeSTAR GXL DNA Polymerase, TaKaRa) in the conditions recommended by the manufacturer ($T°_{annealing}$ = 60°C). Forward primers used for PCR amplification are, respectively, RH_prom208_F, RH_prom508_F, RH_prom1208_F, and RH_prom1508_F; while the common reverse primer is RH-5UTR_R (Table S1). Forward and reverse primers contain, respectively, a KpnI (GGTACC) and a NcoI restriction site

(CCATGG) necessary for the subsequent cloning step (Table S1).

Following PCR amplification, the four inserts were treated enzymatically (Cloning Enhancer, TaKaRa) for 37°C for 15 min, then 80°C for 15 min. The inserts were individually subcloned within the linearized pDP-Empty vector by homologous recombination (In-Fusion HD Cloning Kit, TaKaRa) as indicated above, therefore resulting in the production of the pDP-RH208, pDP-RH508, pDP-RH1208, and pDP-RH1508 plasmid constructs (Figure 1), which were controlled by Sanger sequencing.

Site-directed mutagenesis

The pDP-RH208 and pDP-RH508 vectors both served as templates for generating the variant plasmid constructs containing the c.1-83G>T, c.1-110A>C, and c.1-115A>C variants (Table S2) by site-directed mutagenesis (QuikChange II XL Site-Directed Mutagenesis Kit, Agilent Technologies, Les Ulis, France) following the manufacturer's instructions. All plasmid constructs were controlled by Sanger sequencing before being used for the functional assay.

SUPPLEMENTARY TABLES

TABLE S1. Genotypes of the Thai blood donors carrying at least one reference *RHD* gene copy (i.e. *RHD*01*) and selected for molecular analysis of three additional regions of interest within the gene locus.

**Donor ID**	**D phenotype**[a]	***RHD* genotype**[b]
W003	Weak	**01/*01N.01*
W009	Weak	**01/*01N.01*
W019	Weak	**01/*01N.01*
W022	Weak	**01/*01N.01*
W023	Weak	**01/*01N.03*
W032	Weak	**01/*01N.01*
W034	Weak	**01/*01N.01*
W048	Weak	**01/*01N.01*
W050	Weak	**01/*01*
W051	Weak	**01/*01*
N046	D-negative	**01/*01N.01*
N442	D-negative	**01/*01N.01*
N501	D-negative	**01/*01N.01*
N589	D-negative	**01/*01N.03*
N619	D-negative	**01/*01N.03*
N648	D-negative	**01/*01N.01*
N826	D-negative	**01/*01N.01*
N963	D-negative	**01/*01N.01*

N982	D-negative	**01/*01N.01*
NN061	D-negative	**01/*01N.01*
NN287	D-negative	**01/*01EL.01*
NN415	D-negative	**01/*01N.01*
NN417	D-negative	**01/*01N.01*
NN440	D-negative	**01/*01EL.01*
NN622	D-negative	**01/*01N.01*
NN685	D-negative	**01/*01N.01*
NN732	D-negative	**01/*01N.01*
NN738	D-negative	**01/*01N.01*
RE049	D-negative	**01/*01N.01*
RE094	D-negative	**01/*01EL.01*
RE154	D-negative	**01/*01*
RS224	D-negative	**01/*01N.01*
RS226	D-negative	**01/*01N.01*

[a] Determined by routine serological testing.[1,2]

[b] Determined by extensive molecular analysis.[1,2] Nomenclature in accordance with the recommendations of the ISBT Red Cell Immunogenetics and Blood Group Terminology Working Party (www.isbtweb.org/resource/004rhd.html).

TABLE S2. Primers for PCR amplification and site-directed mutagenesis.

Primer ID	Primer sequence (5'→3')[a]
RVp3br_NotI_F	ATATTTTATTGCGGCCGCCTAGCAAAATAGGCTGTCCC
RVp4br_NotI_R	GGTACTTGGAGCGGCCGCGACGATAGTCATGCCCCGCG
RH_prom208_F	TCTATCGATAGGTACCTAACAGGAACACAGCAACTTG
RH_prom508_F	TCTATCGATAGGTACCGTGGGTCCTATCTGTATCCTCC
RH_prom1208_F	TCTATCGATAGGTACCTCTGATCTACATAGGAATTGTTTTC
RH_prom1508_F	TCTATCGATAGGTACCTCACTAAACAGTCTATCCTCTGTG
RH-5UTR_R	TTGGCGTCTTCCATGGTGTGTCCGTCTCTGTGCAG
RH_c.1-83T_F	GTGTCTCCCCTATCTCTCCCTCAAGCCCT
RH_c.1-83T_R	AGGGCTTGAGGGAGAGATAGGGGAGACAC
RH_c.1-110C_F	AGCCTTGCAGCCTGAGATACGGCCTTTGGC
RH_c.1-110C_R	GCCAAAGGCCGTATCTCAGGCTGCAAGGCT
RH_c.1-115C_F	CAGCCTTGCAGCCTGCGATAAGGCCTTTGGC
RH_c.1-115C_R	GCCAAAGGCCTTATCGCAGGCTGCAAGGCTG

Primers were designed by using the QuikChange Primer Design tool freely available online (URL: www.agilent.com/store/primerDesignProgram.jsp).

[a] The NotI (GCGGCCGC), KpnI (GGTACC), and NcoI (CCATGG) restriction sites are underlined, respectively.

SUPPLEMENTARY FIGURE

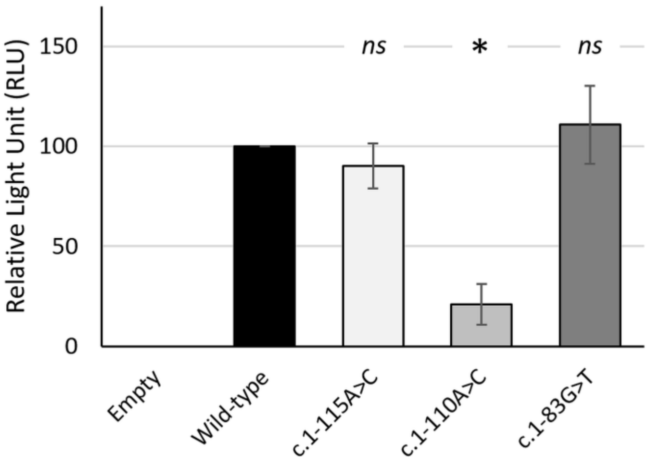

FIGURE S1. Relative transcriptional activity in the presence of three variants in the *RHD* promoter region (reference: pDP-RH208; RLU = 100). Fisher test (N = 4): * $p < 10^{-3}$; *ns*: not significant.

SUPPLEMENTARY REFERENCES

1. Thongbut J, Laengsri V, Raud L, et al. Nation-wide investigation of *RHD* variants in Thai blood donors: impact for molecular diagnostics. Transfusion. 2021;61:931–8.
2. Nuchnoi P, Thongbut J, Bénech C, et al. Serologically D-negative blood donors in Thailand: molecular variants and diagnostic strategy. Blood Transfus. 2023;21:209–17.
